# Hypoxic Pulmonary Hypertension: Molecular Mechanisms and Clinical Research Advances

**DOI:** 10.3390/ijms27062762

**Published:** 2026-03-18

**Authors:** Xiaoyu Fang, Yuanzhou He

**Affiliations:** Department of Respiratory and Critical Care Medicine, Tongji Hospital, Tongji Medical College, Huazhong University of Science and Technology, Wuhan 430030, China; 15307133903@163.com

**Keywords:** hypoxic pulmonary hypertension, hypoxia, molecular mechanisms, clinical research advances

## Abstract

Hypoxic pulmonary hypertension (HPH), classified as Group 3 pulmonary hypertension in the current clinical classification system, represents a complex and progressive cardiopulmonary disorder characterized by elevated pulmonary arterial pressure due to chronic alveolar hypoxia. This condition significantly contributes to morbidity and mortality in patients with chronic lung diseases and individuals residing at high altitudes. The pathogenesis of HPH involves a multifactorial interplay between sustained hypoxic pulmonary vasoconstriction, pulmonary vascular remodeling, endothelial dysfunction, and inflammatory responses. This review provides a comprehensive synthesis of recent advances in HPH pathophysiology and their clinical translation, with a focus on integrating molecular mechanisms with emerging therapeutic strategies. The pathogenesis of HPH involves a complex interplay of hypoxia-inducible factor (HIF) signaling, mechanosensitive ion channel dysregulation (particularly TRPC channels), metabolic reprogramming featuring glycolytic shift and mitochondrial dysfunction, immune–inflammatory mechanisms including macrophage-centered immunopathology, and dysregulation of the nitroxidergic system. Recent clinical advances include refined risk stratification using advanced echocardiographic techniques, identification of novel biomarkers such as lactylation-associated proteins, and development of targeted therapies including immunomodulatory approaches, metabolic modulators, and epigenetic interventions. Ongoing clinical trials are investigating innovative strategies ranging from iron supplementation to nanoparticle-based drug delivery systems. Despite these advances, significant translational challenges remain, including limitations of preclinical models, patient heterogeneity, and the need for HPH-specific outcome measures. This review bridges the gap between mechanistic insights and clinical applications, offering an integrated framework that highlights precision medicine approaches, emerging therapeutic targets, and priority research directions for improving outcomes in this challenging condition.

## 1. Introduction

Hypoxic pulmonary hypertension (HPH) is a progressive vascular disorder characterized by elevated pulmonary arterial pressure resulting from chronic exposure to low-oxygen environments [1]. As defined in the 2022 ESC/ERS guidelines, HPH falls under Group 3 pulmonary hypertension, which encompasses conditions associated with lung diseases and/or hypoxia [2]. This classification distinguishes HPH from other forms of pulmonary hypertension, emphasizing its unique pathogenesis and clinical considerations. The clinical significance of HPH extends beyond high-altitude residents to encompass patients with chronic obstructive pulmonary disease (COPD), interstitial lung diseases, sleep-disordered breathing, and other conditions characterized by alveolar hypoxia [3]. Epidemiological studies suggest that pulmonary hypertension complicates approximately 30–50% of advanced COPD cases, substantially worsening prognosis and quality of life [4].

The pathophysiological cascade of HPH is triggered by sustained hypoxic pulmonary vasoconstriction (HPV), an adaptive mechanism that becomes maladaptive when chronically activated [5]. This initial vasoconstrictive response is followed by structural remodeling of pulmonary arteries, characterized by medial hypertrophy, intimal thickening, and, in advanced stages, plexiform lesion formation. These vascular changes increase pulmonary vascular resistance, imposing pressure overload on the right ventricle and potentially leading to right heart failure [6]. The transition from adaptive HPV to pathological HPH involves complex cellular and molecular interactions among pulmonary vascular cells (endothelial cells, smooth muscle cells, fibroblasts) and immune cells that infiltrate the vascular wall [7].

Despite advances in understanding HPH pathophysiology, therapeutic options remain limited [8]. It is crucial to emphasize that conventional pulmonary vasodilators approved for Group 1 Pulmonary Arterial Hypertension (PAH) have generally shown disappointing results in HPH, and their use in Group 3 patients is not routinely recommended due to the potential risk of worsening ventilation–perfusion mismatch and hypoxemia [9]. This therapeutic gap underscores the need for mechanism-based interventions specifically targeting pathways unique to HPH, rather than simply extrapolating from PAH-directed therapies. Recent years have witnessed significant progress in deciphering the molecular underpinnings of HPH, revealing novel therapeutic targets and diagnostic approaches [10].

**Figure 1:**
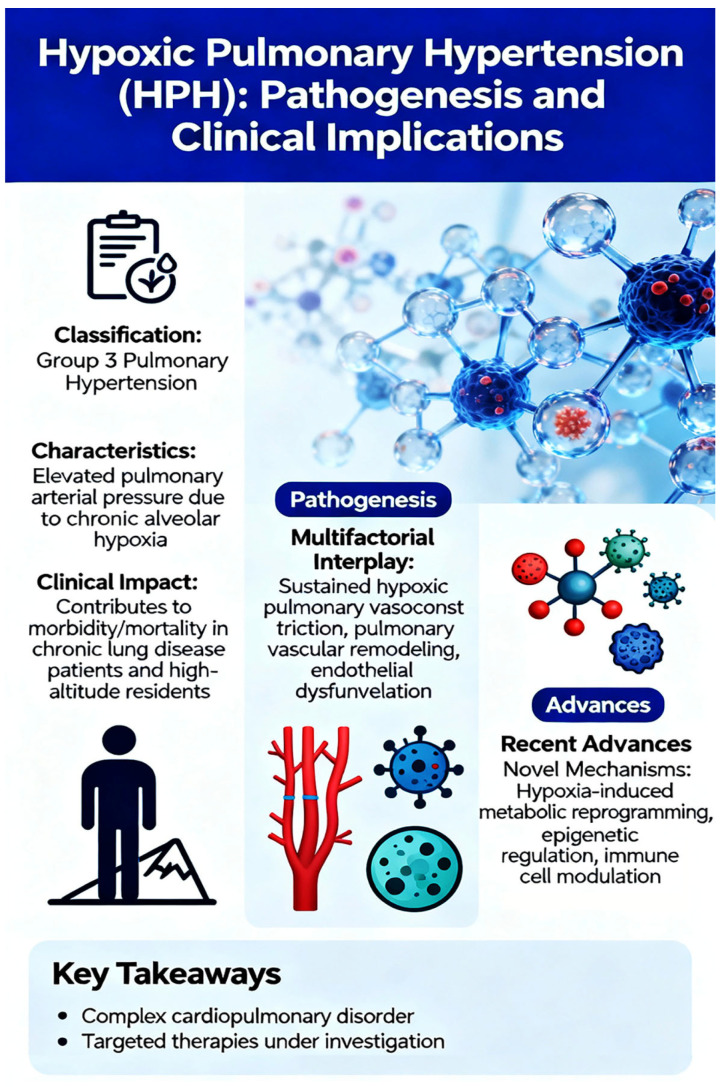
Hypoxic Pulmonary Hypertendion (HPH): Pathogenesis and Clinical Implications.

While several excellent reviews have focused on individual aspects of HPH, such as specific signaling pathways or vascular remodeling [11,12], a significant gap remains in the literature for a truly integrated perspective. Many existing works discuss either the molecular basis or the clinical features in isolation. This review aims to provide a unique and holistic synthesis by explicitly connecting recent breakthroughs in molecular biology—including epigenetics and immunometabolism—with the latest advances in clinical research and therapeutic development. By bridging these traditionally separate domains, we offer a comprehensive framework that not only updates the current understanding of HPH pathogenesis but also directly links mechanistic insights to their potential for clinical translation, an integrative approach that is, to our knowledge, not covered in recent publications. This review aims to synthesize these advances, providing clinicians and researchers with an updated perspective on HPH from molecular mechanisms to clinical applications (Figure 1).

To ensure a balanced and comprehensive review, we conducted a systematic literature search using the PubMed, Web of Science, and Scopus databases. The search strategy was designed to capture literature covering the molecular mechanisms, immunological aspects, and clinical advances in hypoxic pulmonary hypertension. The primary search terms included combinations of ‘hypoxic pulmonary hypertension,’ ‘chronic mountain sickness,’ ‘pulmonary vascular remodeling,’ ‘hypoxia-inducible factors,’ ‘epigenetics,’ ‘inflammation,’ ‘immunometabolism,’ ‘biomarkers,’ and ‘clinical trials’.

Inclusion Criteria: We considered original research articles, comprehensive reviews, and clinical trial reports published in peer-reviewed journals. The search was primarily focused on the last decade (2014–2025) to emphasize recent advances, but landmark older studies were also included for historical context and foundational knowledge.

Exclusion Criteria: Articles not published in English, conference abstracts, and opinion pieces without substantial data were excluded. Studies focusing on pulmonary hypertension etiologies not primarily driven by hypoxia were also excluded to maintain a clear focus on HPH.

## 2. Core Molecular Mechanisms in Hypoxic Pulmonary Hypertension Pathogenesis

### 2.1. Hypoxia-Inducible Factor Signaling Pathways

Central regulators of cellular adaptation to hypoxia and hypoxia-inducible factors (HIFs) play pivotal roles in HPH pathogenesis [13]. HIFs are heterodimeric transcription factors consisting of oxygen-sensitive α subunits (HIF-1α, HIF-2α, HIF-3α) and constitutively expressed β subunits. Under normoxic conditions, HIF-α subunits undergo proteasomal degradation mediated by prolyl hydroxylase domain (PHD) enzymes and the von Hippel–Lindau tumor suppressor protein [14]. During hypoxia, PHD activity decreases, leading to HIF-α stabilization, nuclear translocation, and transcriptional activation of hundreds of target genes involved in angiogenesis, erythropoiesis, glycolysis, and cell survival [15].

The isoform-specific effects of HIFs in HPH pathogenesis are increasingly recognized. Substantial evidence from rodent models indicates that HIF-1α is particularly important in regulating pulmonary vascular remodeling through the induction of growth factors, including platelet-derived growth factor (PDGF) and vascular endothelial growth factor (VEGF) [16,17]. In contrast, HIF-2α appears to have more prominent effects on endothelial function and vasoactive mediator production, a conclusion supported by both genetic mouse models and human high-altitude population studies [18]. Genetic studies using conditional knockout mice have demonstrated that HIF-2α deletion in endothelial cells attenuates hypoxic pulmonary hypertension, while myeloid-specific HIF-1α deletion reduces vascular remodeling, highlighting cell-type specific functions of these transcription factors [19]. However, it should be noted that the majority of these mechanistic insights derive from animal models, and direct evidence in human HPH tissues remains limited, representing an important area for future validation.

Beyond direct transcriptional regulation, HIFs participate in complex cross-talk with other signaling pathways relevant to HPH. HIF-1α interacts with Notch signaling to promote endothelial-to-mesenchymal transition (EndMT), a process contributing to vascular remodeling [20]. Additionally, HIF-1α regulates expression of microRNAs that modulate pulmonary vascular cell phenotypes [21]. The temporal dynamics of HIF activation may influence disease progression, with acute HIF signaling promoting adaptive responses that become maladaptive when chronically sustained [22]. Pharmacological targeting of HIF pathways, particularly through PHD inhibitors, represents an emerging therapeutic strategy [23], though careful modulation is required to avoid systemic effects such as polycythemia.

**Figure 2:**
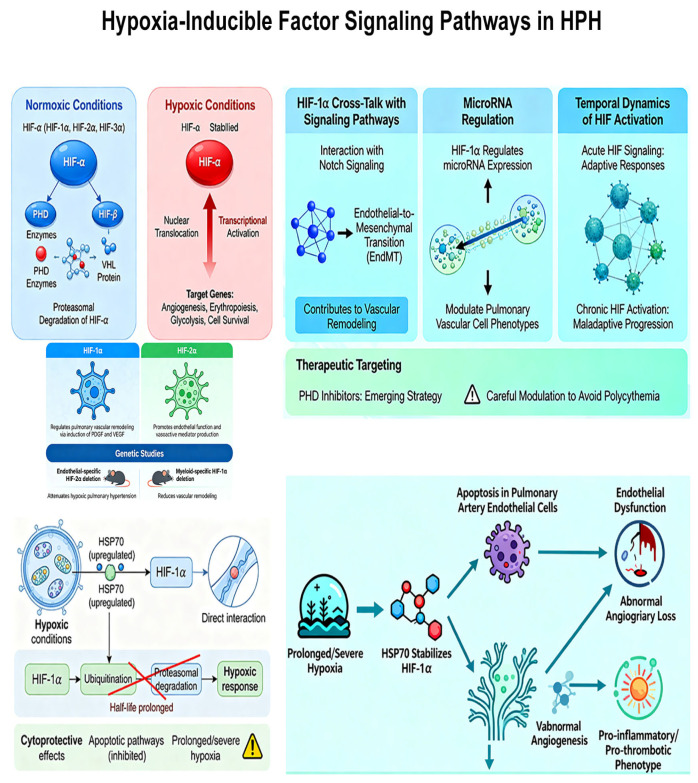
Hypoxia-inducible factor signaling pathways in HPH.

Furthermore, the stability and transcriptional activity of HIF-1α are modulated by molecular chaperones, particularly the 70 kDa heat shock proteins (HSP70) [24]. Under hypoxic conditions, HSP70 expression is upregulated and directly interacts with HIF-1α. This interaction has been shown to prolong the half-life of HIF-1α by preventing its ubiquitination and proteasomal degradation, even under conditions where degradation might otherwise occur. This stabilization amplifies the HIF-1α-mediated hypoxic response. Beyond its chaperone function, HSP70 plays a complex role in cellular fate. It can exert cytoprotective effects by inhibiting key apoptotic pathways [25]. However, in the context of prolonged or severe hypoxia, the sustained stabilization of HIF-1α by HSP70, coupled with other stress signals, can contribute to the initiation of apoptosis in pulmonary artery endothelial cells [26]. This apoptotic process is a critical trigger for endothelial dysfunction, leading to the loss of vascular integrity, abnormal angiogenesis, and the promotion of a pro-inflammatory and pro-thrombotic endothelial phenotype, all of which are fundamental to the pathogenesis of vascular remodeling in hypoxic PH (Figure 2).

### 2.2. Mechanosensitive Signaling and Ion Channel Dysregulation

The biophysical consequences of increased pulmonary vascular resistance and pressure in HPH include elevated mechanical stress on vascular walls, which activates mechanosensitive signaling pathways [27]. Pulmonary artery smooth muscle cells (PASMCs) and endothelial cells express various mechanosensors that convert mechanical stimuli into biochemical signals, including transient receptor potential (TRP) channels, Piezo channels, and integrin-based adhesion complexes [28,29,30]. These mechanosensors play critical roles in the transition from acute vasoconstriction to chronic vascular remodeling.

Among mechanosensitive ion channels, TRP channels have emerged as particularly important mediators of HPH pathogenesis. Preclinical studies using rodent models of chronic hypoxia have demonstrated increased expression and activity of TRP canonical (TRPC) channels, especially TRPC1 and TRPC6, in hypoxic PASMCs [31]. TRPC1 upregulation contributes to enhanced store-operated calcium entry, promoting PASMC proliferation and migration. The functional significance of these channels has been confirmed by studies using TRPC1-deficient mice (trpc1−/−) or TRPC1 siRNA administration, which attenuate pulmonary vascular remodeling and reduce right ventricular systolic pressure in hypoxic models [32]. Similarly, TRPC6 channels, activated by diacylglycerol and mechanical stress, facilitate calcium influx that stimulates PASMC contraction and growth [33]. While these findings are robust in animal models, translational studies confirming the role of TRPC channels in human HPH are still in early stages, with limited data from human pulmonary artery samples.

**Figure 3:**
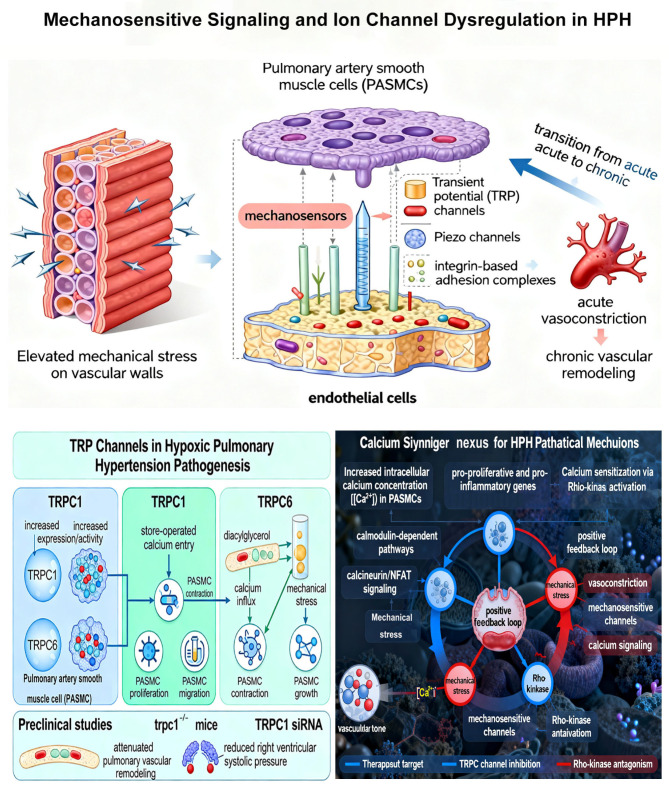
Mechanosensitive signaling and ion channel dysregulation in HPH.

The calcium signaling nexus represents a convergence point for multiple pathological mechanisms in HPH. Increased intracellular calcium concentration ([Ca^2+^]i) in PASMCs activates calmodulin-dependent pathways, including calcineurin/NFAT signaling, which promotes expression of pro-proliferative and pro-inflammatory genes [34,35]. Additionally, calcium sensitization via Rho-kinase activation enhances vascular tone independent of calcium concentration [36]. It is important to note that while Rho-kinase inhibitors have shown efficacy in animal models of HPH and are approved for other cardiovascular conditions in some countries, their specific application in HPH remains investigational, with no large-scale clinical trials completed to date. This signaling integration creates positive feedback loops wherein vasoconstriction increases mechanical stress, further activating mechanosensitive channels and amplifying calcium signaling. Therapeutic targeting of this axis, particularly through TRPC channel inhibition or Rho-kinase antagonism, represents a promising strategy for HPH (Figure 3).

### 2.3. Metabolic Reprogramming in Pulmonary Vascular Cells

Metabolic plasticity represents a fundamental adaptation of pulmonary vascular cells to chronic hypoxia, with significant implications for HPH pathogenesis [37]. Under normoxic conditions, PASMCs primarily utilize oxidative phosphorylation for ATP generation. However, hypoxia induces a metabolic shift toward glycolysis, even in the presence of adequate oxygen—a phenomenon analogous to the Warburg effect in cancer cells [38]. This metabolic reprogramming is well-documented in both animal models and in vitro studies of PASMCs exposed to hypoxia. However, direct evidence from human HPH tissues is more limited, with most human data coming from indirect measurements such as metabolomic profiling of plasma or explanted lung tissue from end-stage patients, which may not reflect early disease stages.

The pentose phosphate pathway (PPP), branching from glycolysis, assumes particular importance in hypoxic PASMCs [39]. Glucose-6-phosphate dehydrogenase (G6PD), the rate-limiting enzyme of the PPP, demonstrates increased activity in hypoxic conditions. G6PD upregulation enhances the production of NADPH, maintaining redox balance and providing reducing equivalents for lipid and nucleotide biosynthesis [40]. Pharmacological inhibition of G6PD or genetic suppression attenuates hypoxia-induced PASMC proliferation and pulmonary vascular remodeling in preclinical models, highlighting this enzyme as a potential therapeutic target. However, these findings remain at the preclinical stage, with no clinical trials of G6PD inhibitors in HPH reported to date. Beyond its metabolic functions, G6PD-derived NADPH supports NADPH oxidase activity, contributing to reactive oxygen species (ROS) generation that further promotes proliferative signaling [41].

Mitochondrial adaptations to chronic hypoxia include both structural and functional alterations [42]. Fragmented mitochondrial networks with reduced cristae density have been observed in PASMCs from HPH models, associated with decreased oxidative capacity and increased ROS production [43]. These dysfunctional mitochondria not only compromise cellular energetics but also release pro-apoptotic factors and damage-associated molecular patterns that promote inflammation. Mitochondrial ROS, particularly superoxide and hydrogen peroxide, serve as signaling molecules that activate HIFs, promote calcium release from intracellular stores, and stimulate pro-inflammatory transcription factors [44]. This creates a vicious cycle wherein mitochondrial dysfunction exacerbates hypoxic signaling while hypoxia further impairs mitochondrial function.

Mitochondrial dysfunction is a cornerstone of the pathophysiology in hypoxic pulmonary hypertension, and it is useful to distinguish between its primary and secondary forms [45]. Primary mitochondrial dysfunction refers to intrinsic abnormalities in mitochondrial components, which can be genetically determined (e.g., mutations in mitochondrial DNA or nuclear genes encoding mitochondrial proteins). While less common as a direct cause, such primary defects can predispose individuals to more severe pulmonary vascular responses to hypoxic stress. More prevalent in the context of hypoxic PH is secondary or acquired mitochondrial dysfunction [46]. This arises as a consequence of the hypoxic environment itself and the associated cellular stress. Chronic hypoxia directly impairs the electron transport chain, particularly at Complex I and IV, leading to decreased ATP production and a metabolic shift towards glycolysis [47]. More critically, this dysfunction results in the overproduction of mitochondrial reactive oxygen species (mtROS), creating a vicious cycle of oxidative damage to mitochondrial lipids, proteins, and DNA. This acquired damage further impairs mitochondrial function, triggers the opening of the mitochondrial permeability transition pore (mPTP), and releases pro-apoptotic factors like cytochrome c, thereby driving endothelial cell apoptosis and smooth muscle cell proliferation [48]. Thus, while a primary defect can be a trigger, the acquired mitochondrial dysfunction driven by chronic hypoxia is a key amplifier of disease pathology in hypoxic PH.

**Figure 4:**
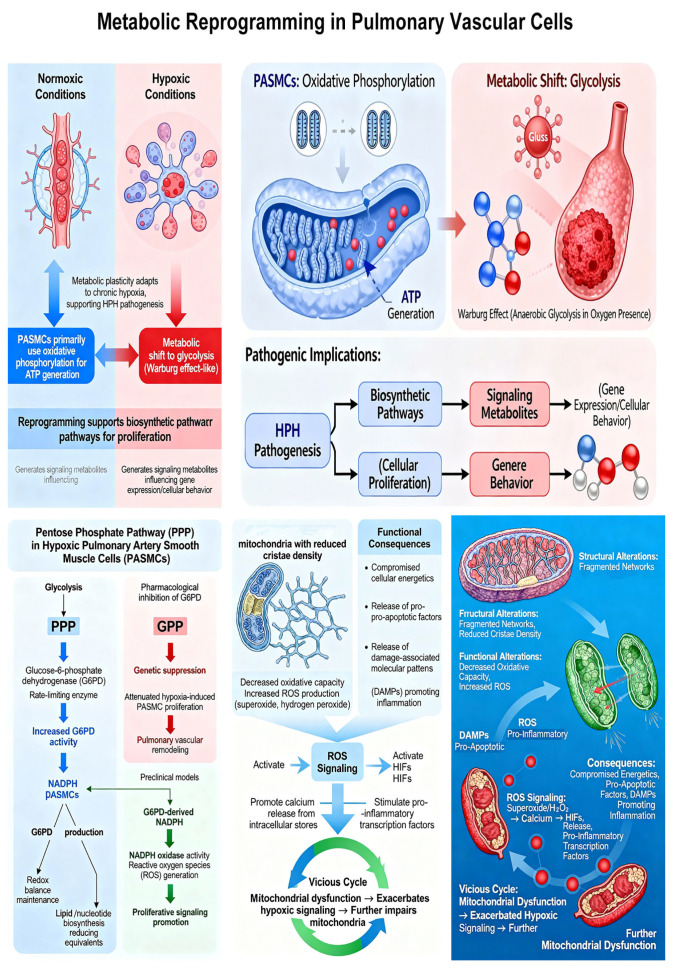
Metabolic reprogramming in pulmonary vascular cells of HPH.

Metabolite signaling extends beyond traditional second messengers to include recently recognized modifications such as protein lactylation. Lactate, accumulating during enhanced glycolysis, can modify lysine residues on histone and non-histone proteins, altering their function [49]. A comprehensive profiling study identified lymphocyte cytosolic protein 1 (LCP1) as a core lactylation-related hub gene significantly upregulated in PH patients and validated in hypoxic PASMCs [50]. This epigenetic–metabolic interface represents a novel dimension of hypoxic signaling, but the evidence is currently limited to correlative studies and in vitro experiments; functional in vivo studies and validation in human HPH cohorts are needed to establish its pathogenic relevance. (Figure 4).

### 2.4. Immune and Inflammatory Mechanisms

Systemic inflammation represents a hallmark of many conditions associated with HPH, particularly COPD and interstitial lung diseases [51]. However, emerging evidence indicates that inflammation is not merely a parallel process but an integral component of HPH pathogenesis [52]. Hypoxia itself exerts direct immunomodulatory effects, activating innate immune responses through pattern recognition receptors and promoting leukocyte recruitment to the pulmonary vasculature [53]. The resulting inflammatory microenvironment contributes to endothelial dysfunction, vascular remodeling, and thrombosis.

Myeloid cell populations, particularly monocytes and macrophages, play crucial roles in HPH progression [51]. Non-classical monocytes (Ly6Clo in mice, CD14^+^CD16^+^ in humans) demonstrate specific responsiveness to hypoxic stimuli and accumulate around remodeled pulmonary vessels in HPH [54,55]. These observations are primarily derived from mouse models and human histopathological studies of explanted lungs, providing strong correlative evidence. Functional studies using myeloid-specific HIF-1α deletion in mice have confirmed that hypoxic signaling in these immune cells is necessary for the full development of HPH [56]. However, the precise mechanisms by which these cells contribute to vascular remodeling in human HPH remain incompletely understood, and therapeutic targeting of monocytes/macrophages has not yet been tested in clinical trials for HPH.

Endothelial–mesenchymal transition (EndMT) represents a specific inflammatory–vascular interface in HPH pathogenesis [57]. Under hypoxic conditions, pulmonary endothelial cells can undergo phenotypic transition to mesenchymal-like cells, losing endothelial markers (VE-cadherin, CD31) while acquiring mesenchymal markers (α-SMA, vimentin). This cellular transdifferentiation contributes to vascular remodeling by increasing the population of mesenchymal cells that produce extracellular matrix and promote vascular stiffness [52]. Multiple signaling pathways regulate hypoxia-induced EndMT, including transforming growth factor-β (TGF-β)/bone morphogenetic protein (BMP) signaling, Notch signaling, and Wnt/β-catenin pathways [58]. EndMT-derived cells exhibit enhanced proliferative and migratory capacities while producing pro-inflammatory mediators that further amplify vascular inflammation.

**Figure 5:**
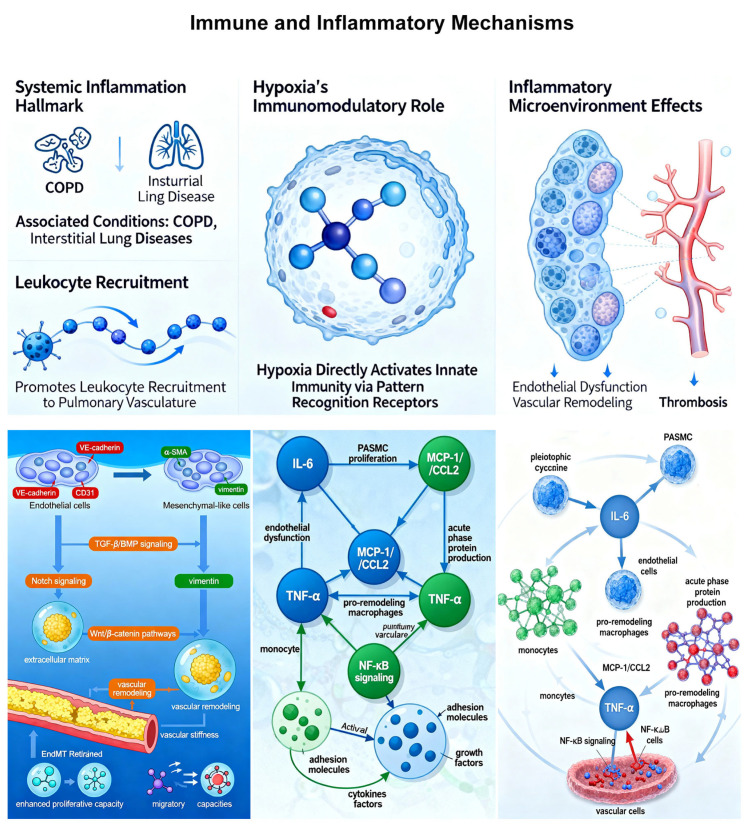
Immune and Inflammatory Mechanisms in HPH.

Cytokine networks in HPH create complex paracrine and autocrine signaling loops. Interleukin-6 (IL-6), a pleiotropic cytokine increased in HPH, promotes PASMC proliferation and endothelial dysfunction while stimulating acute phase protein production [59]. Monocyte chemoattractant protein-1 (MCP-1/CCL2) recruits monocytes to the pulmonary vasculature, facilitating their differentiation into pro-remodeling macrophages [60]. Tumor necrosis factor-α (TNF-α) activates nuclear factor-κB (NF-κB) signaling in vascular cells, inducing expression of adhesion molecules, cytokines, and growth factors (Table 1). While these cytokines are elevated in HPH patients and animal models, it is important to recognize that most evidence is correlative; causal proof of their necessity in human HPH is lacking. Several immunomodulatory approaches are under investigation in preclinical models, but none have yet advanced to clinical trials specifically for HPH. (Figure 5).

### 2.5. Dysregulation of the Nitroxidergic System

A consistent finding in both experimental models and patients with HPH is decreased NO bioavailability [61]. M Ozaki demonstrated that eNOS expression is diminished in the vascular endothelium of pulmonary arteries from patients with pulmonary hypertension [62]. Subsequent studies have confirmed that chronic hypoxia reduces eNOS expression and activity in the pulmonary circulation, although some conflicting results exist regarding the direction and magnitude of these changes [63]. These discrepancies may reflect temporal dynamics, species differences, or the stage of disease at which measurements are obtained.

The functional consequence of reduced NO bioavailability is the loss of vasoprotective mechanisms, including impaired endothelium-dependent vasodilation, enhanced smooth muscle proliferation, and increased susceptibility to vasoconstrictor stimuli [64]. In animal models, chronic hypoxia leads to blunted NO-mediated relaxation of pulmonary arteries, correlating with the development of pulmonary hypertension [65].

Paradoxically, even when eNOS protein levels are maintained or increased, the enzyme may become “uncoupled”, which is a state in which the electron flow through the reductase domain is diverted from L-arginine oxidation to molecular oxygen, resulting in superoxide (O_2_•^−^) production rather than NO [66]. This phenomenon occurs under conditions of BH4 deficiency, L-arginine depletion, or oxidative stress, all of which may be present in the hypoxic pulmonary circulation [67]. The concept of eNOS uncoupling in HPH is supported by preclinical studies and indirect human data, but direct evidence of uncoupling in human HPH vasculature remains limited.

The superoxide generated by uncoupled eNOS rapidly reacts with any remaining NO to form peroxynitrite (ONOO^−^), a potent oxidant that further depletes BH4 and exacerbates eNOS uncoupling, establishing a vicious cycle. Peroxynitrite modifies proteins through nitration of tyrosine residues, with 3-nitrotyrosine serving as a footprint of this process in hypertensive pulmonary vessels [68]. These oxidative and nitrative modifications impair the function of multiple proteins, including sGC and PKG, amplifying the inhibitory effect associated with NO inactivation [61].

The hypoxic pulmonary environment is characterized by increased production of reactive oxygen species (ROS) from multiple sources. NADPH oxidases (NOX enzymes), particularly NOX1 and NOX4, are upregulated in the hypertensive pulmonary vasculature and generate superoxide that scavenges NO [69]. Mitochondrial dysfunction under hypoxia also contributes to ROS production through electron transport chain abnormalities [70]. The resulting increase in oxidative stress not only consumes NO but also promotes vasoconstriction and vascular remodeling through direct effects on smooth muscle cells.

Downstream of NO production, the sGC/cGMP pathway is also compromised in HPH. Oxidative stress can oxidize the heme iron of sGC, rendering the enzyme insensitive to NO stimulation. Additionally, peroxynitrite-mediated nitration of PKG may reduce its activity, diminishing the cellular response to cGMP [71]. These downstream defects explain why simply increasing NO availability may be insufficient to restore normal vascular function in established disease and provide the rationale for therapeutic strategies that bypass these abnormalities. It is worth noting that while sGC stimulators are approved for PAH and chronic thromboembolic PH, their efficacy in HPH has not been established in dedicated trials, and current guidelines do not recommend their routine use in Group 3 PH. Hypoxia-inducible factor-1α (HIF-1α) plays a central role in the transcriptional response to chronic hypoxia and interacts with the nitroxidergic system at multiple levels. HIF-1α activation promotes the expression of genes involved in vasoconstriction, inflammation, and remodeling, while also influencing NOS expression and function [72]. The relationship between HIF-1α and NO signaling is bidirectional, as NO can modulate HIF-1α stability through effects on prolyl hydroxylase activity. This complex interplay contributes to the integrated vascular response to hypoxia and represents a potential therapeutic target [61].

### 2.6. Integration of Mechanistic Pathways in HPH Progression

The progression of HPH is therefore a vicious cycle where Hypoxia (via HIF) initiates vascular remodeling. This remodeling alters the mechanical environment (mechanotransduction) and cellular metabolism, both of which trigger and sustain immune cell infiltration. In turn, immune responses secrete mediators that make the vascular cells more sensitive to hypoxia and mechanical stress, locking the pathology into a self-perpetuating cycle of obliterative vascular remodeling.

**Figure 6:**
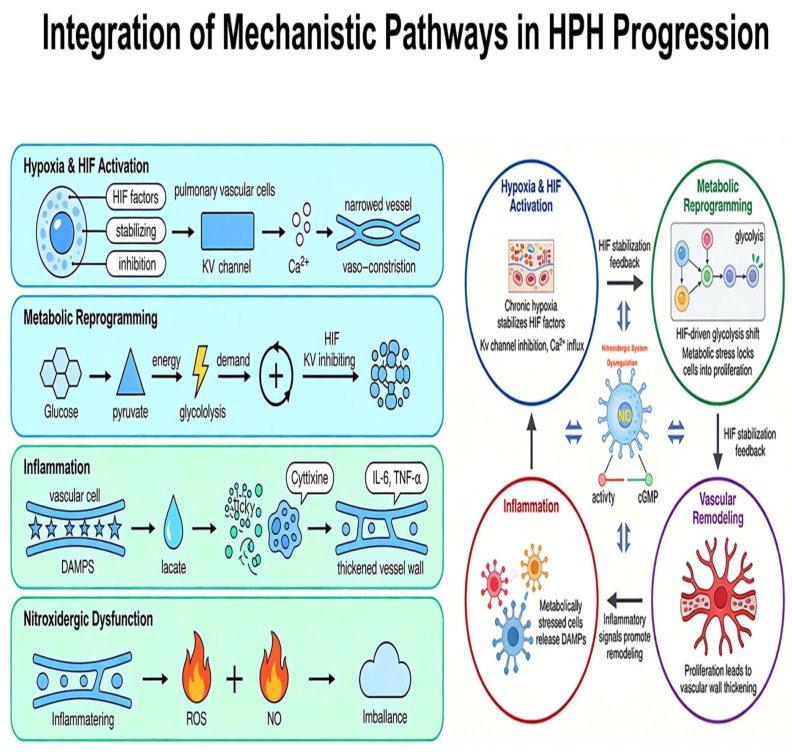
Integration of Mechanistic Pathways in HPH Progression.

Chronic hypoxia stabilizes the Hypoxia-Inducible Factor (HIF) pathway, particularly HIF-1α and HIF-2α, in pulmonary arterial smooth muscle cells (PASMCs) and endothelial cells (PAECs). HIF activation directly triggers the downregulation and dysfunction of voltage-gated potassium channels (Kv), a key component of mechanosensitive and ion channel dysregulation. This inhibits potassium efflux, leading to cell membrane depolarization. Depolarization opens voltage-gated calcium channels, increasing intracellular calcium. This acute mechanism of hypoxic pulmonary vasoconstriction (HPV) is the initial insult. Concurrently, HIF-mediated downregulation of other mechanosensitive channels, like certain transient receptor potential (TRP) channels, alters the cell’s response to mechanical stretch from increased pressure, shifting them toward a proliferative and contractile phenotype. Thus, the biochemical sensor (HIF) directly controls the ion channel machinery that governs the initial vascular response (Figure 6).

The sustained rise in intracellular calcium resulting from ion channel dysfunction places a high energy demand on PASMCs to maintain calcium homeostasis and support contraction. This energy demand fuels the metabolic reprogramming already initiated by HIF. HIF promotes a shift from oxidative phosphorylation to glycolysis (the “Warburg effect”) in pulmonary vascular cells, even in the presence of oxygen. This glycolytic switch is less efficient at producing ATP, forcing the cell to increase glucose uptake and consumption. The resulting mitochondrial dysfunction is crucial: it reduces the production of reactive oxygen species (ROS) from the electron transport chain, which further stabilizes HIF and inhibits Kv channels, creating a powerful positive feedback loop. The cell’s altered metabolism is both a consequence and a driver of its dysfunctional ion channel state.

The metabolic reprogramming of vascular cells directly contributes to a pro-inflammatory environment, integrating with immune and inflammatory mechanisms. Glycolytic PASMCs and PAECs release damage-associated molecular patterns (DAMPs) and metabolites like lactate. Lactate, via the GPR81 receptor, can promote an inflammatory phenotype in macrophages. Concurrently, the hypoxic and metabolically stressed endothelium upregulates adhesion molecules (e.g., VCAM-1, ICAM-1), facilitating the recruitment of circulating immune cells, particularly macrophages and monocytes. Once recruited, these immune cells (e.g., perivascular macrophages) themselves undergo metabolic reprogramming, further amplifying the local inflammatory cytokine cascade (IL-6, TNF-α), which in turn stimulates PASMC proliferation and vascular remodeling.

The inflammatory milieu profoundly exacerbates the dysregulation of the nitroxidergic system. Pro-inflammatory cytokines and oxidative stress from activated immune cells and vascular cells increase the production of reactive oxygen species (ROS), such as superoxide. Superoxide rapidly scavenges nitric oxide (NO) in a diffusion-limited reaction, producing the potent oxidant peroxynitrite. This process causes “endothelial dysfunction” by functionally depleting bioavailable NO, even if endothelial NO synthase (eNOS) expression is unchanged or increased. Peroxynitrite itself can uncouple eNOS, turning it from a NO-producing enzyme into a superoxide-producing one, further worsening the situation. The loss of the vasodilatory and anti-proliferative effects of NO, combined with the direct pro-proliferative effects of inflammation, creates a powerful drive for vascular remodeling.

In summary, HPH progression is driven by the intricate integration of these pathways. HIF signaling initiates the process by altering ion channel function and metabolism. This dysfunctional state creates a pro-inflammatory environment and disrupts NO signaling. In turn, inflammation and nitroxidergic dysfunction feedback to exacerbate the initial triggers (e.g., inflammation can stabilize HIF), creating a self-perpetuating cycle of vasoconstriction, hyperproliferation, and irreversible vascular remodeling that characterizes established HPH.

## 3. Clinical Research Advances

### 3.1. Risk Assessment and Stratification

Accurate risk stratification is essential for guiding therapeutic decisions and predicting outcomes in HPH [73]. Traditional risk assessment tools, derived primarily from studies on pulmonary arterial hypertension (Group 1), have limitations when applied to HPH. Recent efforts have focused on developing HPH-specific parameters that better reflect disease severity and prognosis. Advanced echocardiographic techniques have emerged as particularly valuable non-invasive tools for comprehensive hemodynamic and functional assessment [8].

In a large observational study presented at the 2025 American Heart Association Scientific Sessions, the prognostic value of left ventricular diastolic function assessed by speckle-tracking echocardiography was demonstrated in a cohort of patients with precapillary pulmonary hypertension, including a subset with Group 3 PH [74]. The left ventricular global early diastolic peak strain rate showed significant correlation with exercise capacity (peak oxygen consumption) and N-terminal pro-brain natriuretic peptide (NT-proBNP) levels. More importantly, this parameter predicted all-cause mortality with an area under the curve of 0.689, providing incremental prognostic information beyond conventional right ventricular parameters. While promising, these findings require validation in larger, prospective HPH-specific cohorts before clinical implementation.

Three-dimensional echocardiography has further enhanced risk stratification by enabling accurate volumetric assessment of the right ventricle—a chamber notoriously difficult to image due to its complex geometry [75]. Studies have established that right ventricular end-diastolic volume > 150 mL and end-systolic volume > 109 mL independently predict right heart failure-related mortality in precapillary pulmonary hypertension patients. These volumetric thresholds demonstrated superior specificity compared to the 2015 ESC risk stratification strategy, potentially allowing more precise identification of high-risk patients who may benefit from intensified monitoring and therapy. However, it should be noted that these cutoffs were derived from mixed PH populations and have not been specifically validated in HPH.

### 3.2. Novel Biomarkers and Diagnostic Approaches

The search for reliable biomarkers reflecting PH activity and treatment response continues to be an active area of investigation. Beyond established markers like NT-proBNP, recent studies have explored molecules related to the specific pathogenic processes in PH [76]. Metabolomic profiling has revealed distinct metabolic signatures in HPH patients, with alterations in glycolysis, TCA cycle intermediates, and lipid metabolites [77]. These findings are based on small, cross-sectional studies (early-phase clinical studies, Phase I/II trials and observational studies) and require validation in larger longitudinal cohorts to determine their utility as biomarkers of disease progression or treatment response.

Molecular imaging approaches targeting specific pathways activated in HPH hold promise for non-invasive assessment of disease activity. Positron emission tomography (PET) using radiotracers for glycolysis (^18^F-FDG) or proliferation (^18^F-fluorothymidine) can visualize metabolic and cellular changes in the pulmonary vasculature and right ventricle [78] (early-phase clinical studies Phase I/II trials and observational studies). Preliminary studies suggest that ^18^F-FDG uptake in the right ventricle correlates with right ventricular function and prognosis in pulmonary hypertension, potentially serving as a marker of right ventricular metabolic adaptation to pressure overload. However, these are small single-center studies, and standardization of imaging protocols is needed before clinical adoption. Genetic and epigenetic markers are also being explored for risk prediction and personalized management of HPH [79]. While HPH is generally considered an acquired condition, genetic polymorphisms influencing hypoxic responses may modify individual susceptibility (preclinical evidence, animal models and in vitro studies). Additionally, circulating microRNAs and DNA methylation patterns associated with HPH have been identified, potentially serving as minimally invasive biomarkers reflecting disease activity and treatment response [80] (preclinical evidence, animal models and in vitro studies). These findings are preliminary and have not yet been translated into clinically useful tests.

### 3.3. Therapeutic Strategies and Clinical Translation

Current therapeutic approaches for HPH primarily focus on treating the underlying lung disease and providing supplemental oxygen to correct hypoxemia [81]. This is the standard of care, supported by clinical guidelines based on observational studies and expert consensus. However, disease-modifying therapies specifically targeting pulmonary vascular remodeling are urgently needed. Several novel strategies emerging from mechanistic studies are progressing toward clinical evaluation (Table 2).

Immunomodulatory approaches represent a promising frontier in HPH therapy. Based on preclinical evidence of macrophage-centered immunopathology, early-phase clinical trials are exploring strategies to modify pulmonary immune responses in HPH. These include macrophage-targeting therapies using pharmacological agents that promote anti-inflammatory macrophage polarization or cell-based therapies utilizing engineered regulatory macrophages [82]. It is important to emphasize that these are early-phase trials (Phase I/II) with small sample sizes, primarily assessing safety and proof-of-concept. Their efficacy in HPH remains to be established in larger randomized controlled trials. Metabolic modulators targeting the glycolytic shift in pulmonary vascular cells are another emerging therapeutic class. Preclinical studies with G6PD inhibitors and PFKFB3 modulators have demonstrated efficacy in attenuating pulmonary hypertension in animal models [83,84]. These findings are exclusively preclinical at this stage; no clinical trials of these agents have been initiated in HPH patients. The context-dependent effects of these metabolic interventions—particularly the differential response under normoxic versus hypoxic conditions—highlight the importance of careful patient selection and monitoring in clinical trials. Combination approaches simultaneously targeting multiple metabolic pathways may offer enhanced efficacy while minimizing compensatory adaptations.

Epigenetic therapies represent a novel strategy for modifying gene expression patterns driving pulmonary vascular remodeling. Small molecule inhibitors targeting methyltransferases like SMYD2 have shown promise in preclinical models of HPH [84]. Additionally, RNA-based therapeutics modulating m6A methylation or specific microRNAs involved in HPH pathogenesis are under investigation [85]. All of these approaches are at the preclinical stage, with no clinical trials reported to date. As epigenetic modifications are potentially reversible, these approaches offer the advantage of modifying disease progression without permanent genetic alterations.

### 3.4. Ongoing Clinical Trials and Emerging Therapies

The clinical trial landscape for HPH is evolving, with several innovative studies currently underway. The ICAROS-PH trial (DRKS00028924) represents a unique physiological investigation examining the effects of hypobaric hypoxia (simulating long-haul flight conditions) on patients with various forms of pulmonary hypertension [86]. This prospective, crossover study comparing 8 h of hypobaric hypoxia to normobaric normoxia aims to establish evidence-based recommendations regarding fitness to fly for pulmonary hypertension patients. Secondary outcomes include comprehensive assessment of cardiopulmonary adaptation, inflammatory biomarkers, and cognitive function during hypoxia exposure. As a physiological study, it will provide valuable data but is not designed to test therapeutic interventions.

Another notable trial investigates the effects of high-concentration oxygen (100% via Conoxia^®^, Linde AG, Unterschleißheim, Germany) on pulmonary and coronary hemodynamics in patients with pulmonary hypertension, coronary microvascular dysfunction, hypoxia, or heart failure [87]. This study, expected to complete in October 2025, is a mechanistic investigation of acute hemodynamic responses. It will provide insights into oxygen therapy optimization but does not represent a chronic treatment trial.

Several early-phase trials are evaluating targeted therapies based on recently elucidated molecular mechanisms. These include a Phase II study of iron supplementation in iron-deficient pulmonary hypertension patients (including those with Group 3 PH), based on evidence linking iron homeostasis to mitochondrial function in HPH [88]. Other early-phase trials are exploring repurposed medications with potential benefits in HPH, such as PPARγ agonists based on their ability to counteract SMYD2-mediated effects [89]. The outcomes of these small, proof-of-concept trials will determine whether mechanistic insights from basic research can translate to clinical benefits and will inform the design of larger definitive studies (Table 3).

## 4. Future Directions and Translational Challenges

### 4.1. Precision Medicine Approaches in HPH

The heterogeneous nature of HPH across different etiologies and individual patients necessitates a precision medicine approach to optimize diagnosis and treatment. Emerging technologies, particularly multi-omics profiling, offer unprecedented opportunities to characterize HPH subtypes based on molecular signatures. Integration of genomic, transcriptomic, proteomic, metabolomic, and epigenomic data from well-phenotyped patient cohorts may identify distinct HPH endotypes with differential therapeutic responsiveness [90]. For example, transcriptomic analyses have already revealed subsets of HPH patients with prominent inflammatory signatures versus those with predominant metabolic dysregulation, suggesting the potential for tailored therapeutic approaches. However, these findings are still in the discovery phase and require validation in independent cohorts before clinical application.

Biomarker discovery represents a critical component of precision HPH management. Beyond established biomarkers like BNP and NT-proBNP, novel candidates reflecting specific pathogenic processes are needed. Lactylation-associated proteins, including LCP1 identified in recent proteomic studies, show promise as biomarkers reflecting metabolic reprogramming in HPH [50]. Circulating extracellular vesicles containing cell-specific cargo may provide non-invasive windows into pulmonary vascular pathology. Additionally, imaging biomarkers beyond standard echocardiographic parameters, including right ventricular strain analysis, pulmonary artery stiffness measurements, and molecular imaging of specific pathways, could enhance disease characterization and treatment monitoring [91]. All of these candidate biomarkers are at an early stage of development, with most studies being small and exploratory. Prospective validation in large cohorts is needed.

Genetic predisposition to HPH development and progression represents another dimension of precision medicine [92]. While most HPH cases are considered acquired rather than heritable, genetic variants may modify individual susceptibility to hypoxic vascular remodeling. Genome-wide association studies in high-altitude populations have identified candidate loci associated with adaptation to chronic hypoxia, some of which involve pulmonary vascular regulation [93]. Furthermore, somatic mutations similar to those observed in PAH, such as BMPR2, ACVRL1, SMAD9, have been identified in some severe HPH cases, suggesting potential overlap in pathogenic mechanisms, but the role of hypoxia-induced epigenetic regulation and dysregulation of the BMPR2 pathway in HPH remains distinct. Genetic screening in selected HPH patients, particularly those with severe or early-onset disease, may identify therapeutic targets and inform family counseling. These genetic findings are primarily from population studies and case series; their utility for individual risk prediction in clinical practice remains to be established.

In the context of high-altitude dwellers, it is pertinent to consider whether the pathophysiological responses to hypobaric hypoxia (as experienced at altitude) differ from those to normobaric hypoxia (as in chronic lung disease). While the primary stimulus—a decrease in alveolar PO_2_—is similar, the physiological responses may not be entirely equivalent. Hypobaric hypoxia exposes individuals to not only lower partial pressure of oxygen but also reduced barometric pressure, which can affect respiratory mechanics and diffusing capacity. Some studies suggest that for a given inspired PO_2_, hypobaric hypoxia may elicit a greater degree of sympathetic activation and potentially more pronounced pulmonary vascular responses compared to normobaric hypoxia, possibly due to differences in ventilatory patterns or fluid regulation. However, the long-term consequences, such as the development of severe pulmonary hypertension and right heart failure in maladapted individuals, appear to be convergent, driven by the sustained pressure overload from hypoxic pulmonary vasoconstriction and subsequent vascular remodeling. This distinction is crucial for translating findings from animal studies (which typically use normobaric hypoxia) to human populations at altitude and vice versa, and highlights the need for careful consideration of experimental conditions when interpreting preclinical data [5].

### 4.2. Innovative Therapeutic Targets and Delivery Systems

The limited efficacy of systemic drug administration in HPH, due to poor pulmonary vascular targeting and systemic side effects, has spurred development of innovative delivery approaches. Inhaled formulations of vasodilators represent an established strategy to enhance pulmonary specificity, but newer nanotechnology platforms offer further advantages. Polymeric nanoparticles, liposomes, dendrimers, and extracellular vesicles can be engineered for pulmonary vascular targeting through size optimization or surface modification with targeting ligands.

Stimuli-responsive systems represent particularly promising approaches for HPH [94,95]. Nanoparticles that release their payload in response to the HPH microenvironment—such as low pH, elevated reactive oxygen species, or specific enzyme activities—could provide spatiotemporal control of drug delivery. For example, ROS-responsive nanoparticles releasing therapeutic agents specifically in areas of vascular oxidative stress could target pathological signaling while sparing normal vasculature. Similarly, enzyme-activated prodrugs activated by matrix metalloproteinases or other enzymes upregulated in vascular remodeling offer another approach for localized therapy.

Combination strategies targeting multiple pathogenic pathways simultaneously may overcome the limitations of single-agent approaches. Nanocarriers co-delivering a vasodilator, an anti-proliferative agent, and an anti-inflammatory drug could address the multifactorial nature of HPH progression. Furthermore, sequential release systems that deliver different agents with distinct kinetics could mimic optimal dosing regimens while simplifying administration. It is important to note that all of these nanotechnology-based approaches are currently at the preclinical stage, with no clinical trials in HPH patients reported to date. Rational design of such combination approaches requires improved understanding of pathway interactions and temporal relationships in HPH pathogenesis.

Recent advances have not only refined our understanding of hypoxic PH but have also illuminated a new generation of therapeutic targets. The central pathogenic pathways discussed in this review, namely redox imbalance (encompassing both reactive oxygen species (ROS) and reactive nitrogen species (RNS)), and the resulting endothelial dysfunction, are now recognized as critical intervention points. Innovations in the field are increasingly focused on precisely modulating these components. For example, therapies are being developed to specifically target mitochondrial ROS production, aiming to restore redox homeostasis without disrupting essential physiological ROS signaling [96]. Concurrently, strategies to reverse endothelial dysfunction are moving beyond simple vasodilation to include agents that promote endothelial barrier integrity, reduce apoptosis, and correct the imbalanced production of vasoactive factors like NO and endothelin-1. Furthermore, targeting the intricate crosstalk between these components—such as how ROS can uncouple endothelial nitric oxide synthase (eNOS) to exacerbate both oxidative and nitrosative stress—represents a sophisticated, multi-pronged approach. These specific targets offer the potential for disease-modifying therapies that go beyond symptomatic relief to directly address the underlying vascular pathology, but they remain largely in the preclinical or very early clinical development phase.

### 4.3. Advanced Preclinical Models and Translational Gaps

Model limitations have historically constrained HPH research, with most preclinical studies utilizing simple chronic hypoxia exposure in rodents. While these models reproduce aspects of human HPH, they lack the complexity of concomitant lung disease that characterizes most clinical cases. Development of combinatorial models incorporating both hypoxia and lung injury may better recapitulate human disease pathophysiology. Additionally, large animal models with pulmonary anatomy and physiology more similar to humans could improve translational predictability, though with increased cost and ethical considerations.

Organoid systems and microphysiological models (“lung-on-a-chip”) offer innovative platforms for HPH research. Pulmonary vascular organoids derived from human-induced pluripotent stem cells (iPSCs) containing endothelial cells, smooth muscle cells, and pericytes in three-dimensional architecture can model cell–cell interactions in vascular remodeling. Microfluidic devices recreating pulmonary capillary networks with controlled oxygen tension and mechanical forces enable the study of hemodynamic effects on vascular biology. These human-based systems complement animal studies and may be particularly valuable for personalized medicine approaches using patient-derived cells.

The translational pathway from preclinical discovery to clinical application in HPH faces several specific challenges. First, the heterogeneous patient population with different underlying lung diseases complicates trial design and interpretation. Second, regulatory pathways for HPH therapies are less established than for PAH, creating uncertainty for drug development. Third, outcome measures for clinical trials require refinement, as traditional PAH endpoints may not fully capture benefits in HPH. Addressing these challenges will require collaborative efforts among basic scientists, clinical researchers, pharmaceutical developers, regulatory agencies, and patient advocates to establish optimized pathways for HPH therapeutic development.

### 4.4. Unanswered Questions and Research Priorities

Despite significant advances, fundamental questions regarding HPH pathogenesis and treatment remain unanswered. The temporal sequence of molecular events initiating and sustaining pulmonary vascular remodeling in hypoxia requires further elucidation. It is importan to understand why some individuals develop severe HPH while others with similar hypoxic exposure may not reveal critical protective mechanisms. The relative contributions of different cell types (endothelial cells, smooth muscle cells, fibroblasts, immune cells) to vascular remodeling at various disease stages need clarification to guide cell-specific therapeutic targeting.

Sex differences in HPH represent an understudied area with potential therapeutic implications. While PAH demonstrates female predominance, epidemiological data regarding sex distribution in HPH are less clear, though some studies suggest worse outcomes in males. Hormonal influences, sex chromosome effects, and sociocultural factors may all contribute to observed differences. Elucidation of sex-specific mechanisms could identify novel therapeutic targets and inform personalized treatment approaches.

The relationship between aging and HPH susceptibility represents another important research direction. Age-associated changes in vascular stiffness, mitochondrial function, and regenerative capacity may exacerbate hypoxic vascular remodeling. Conversely, cellular senescence in the pulmonary vasculature may limit proliferative responses to hypoxia. Understanding these age–vascular interactions could lead to therapies targeting fundamental aging processes in HPH, particularly relevant given the aging global population.

Finally, the interorgan communication between the remodeled pulmonary vasculature and other organs, particularly the right ventricle, requires deeper investigation. While right ventricular adaptation to increased afterload is recognized as a critical determinant of outcomes in HPH, the molecular signals mediating this adaptation and eventual decompensation remain poorly understood. Similarly, lung–kidney and lung–liver interactions in HPH may contribute to fluid retention and metabolic disturbances observed in advanced disease. A systems biology approach integrating multiple organ systems will be essential for comprehensive understanding and effective treatment of HPH.

Several important limitations should be acknowledged when considering the translational potential of current preclinical findings in hypoxic pulmonary hypertension (HPH). First, the controlled and simplified nature of existing animal models—typically involving chronic exposure to normobaric or hypobaric hypoxia in genetically identical strains—fails to recapitulate the complex, multifactorial pathophysiology of clinical HPH, which often involves comorbidities, genetic diversity, and variable exposure patterns. Second, HPH in humans is a heterogeneous condition, influenced by the underlying cause of hypoxia (e.g., COPD, interstitial lung disease, or sleep-disordered breathing) and individual patient factors, making it unlikely that a single therapeutic target identified in the laboratory will be uniformly effective across all patient subsets. Finally, the field is constrained by a paucity of large-scale, multicenter clinical trials dedicated specifically to HPH. Much of the existing clinical evidence is derived from subgroup analyses of trials focused on pulmonary arterial hypertension (PAH), or from small, single-center studies with limited statistical power. Collectively, these factors create a significant evidence gap, limiting our ability to validate preclinical discoveries in real-world patient populations and hindering the development of targeted, evidence-based treatment guidelines. This underscores the critical need for dedicated HPH research programs that integrate robust preclinical models with well-designed clinical trials.

## 5. Conclusions

Hypoxia-induced pulmonary hypertension represents a complex vascular disorder arising from the interplay of hypoxic signaling, mechanical stress, metabolic reprogramming, and inflammatory activation. The molecular pathogenesis of HPH involves a multilayered network of pathways, with hypoxia-inducible factors serving as master regulators that coordinate cellular responses to low oxygen tension. Based on the current evidence hierarchy, HIF signaling emerges as the most upstream and best-validated driver, while other pathways—such as mechanotransduction, metabolic shifts, immune activation, and nitroxidergic dysfunction—act as critical amplifiers and downstream effectors. This pathogenic complexity explains the limited efficacy of traditional pulmonary vasodilators in HPH and underscores the need for targeted therapeutic approaches.

Clinical management of HPH remains challenging, with current strategies focused primarily on treatment of underlying lung diseases, correction of hypoxemia, and judicious application of PAH-specific therapies in selected patients. It is essential to distinguish between established clinical practices (e.g., oxygen therapy, treatment of underlying lung disease) and investigational approaches. Emerging therapeutic strategies targeting novel molecular pathways, advanced drug delivery systems (particularly nanoparticle-based approaches), and immunomodulatory interventions offer promise for more effective treatment, but most remain at the preclinical or early clinical stage. The development of precision medicine approaches based on molecular subtyping and biomarker-guided therapy represents a particularly promising direction for improving patient outcomes.

Translational research bridging basic discovery and clinical application faces several challenges, including limitations of preclinical models, heterogeneity of the patient population, and regulatory uncertainties. Addressing these challenges will require collaborative efforts across disciplines and sectors, with the integration of advanced technologies such as multi-omics profiling, organoid systems, and computational modeling. As our understanding of HPH pathophysiology deepens and novel therapeutic strategies emerge, there is renewed hope for effectively managing this challenging condition and improving the lives of affected patients worldwide.

## Figures and Tables

**Table 1:** Key Molecular Pathways in Hypoxic Pulmonary Hypertension.

Pathway Category	Key Molecules	Cellular Effects	Therapeutic Implications
Metabolic Reprogramming	G6PD, PFKFB3, HIFs	Enhanced glycolysis, PPP activation, cellular proliferation	G6PD inhibitors, PFKFB3 modulators
Epigenetic Regulation	SMYD2, METTL3, m6A RNA methylation	Altered gene expression, enhanced proliferation, inhibited apoptosis	SMYD2 inhibitors, METTL3 modulators
Post-Translational Modification	eNOS, CaM, Cav-1	Reduced NO bioavailability, increased oxidative stress	eNOS recouplers, Cav-1 stabilizers
Iron Metabolism	HIF-2α, mitochondrial complexes I/III, ROS	Mitochondrial dysfunction, metabolic shift	Iron supplementation, mitochondrial antioxidants
Immune Modulation	M2regs macrophages, inflammatory cytokines	Vascular inflammation, immune cell recruitment	Cell therapy, cytokine modulation

**Table 2:** Summary of Key Emerging Molecular Targets in HPH.

Target/Pathway	Primary Cell Type	Proposed Mechanism in HPH	Potential Therapeutic Intervention
HIF-2α	Endothelial Cells	Disrupts mitochondrial function increases ROS production	Selective HIF-2α inhibitors (e.g., PT2385)
G6PD	PASMCs	Fuels PPP, provides NADPH and precursors for proliferation	G6PD inhibitors (e.g., 6-AN)
xCT (SLC7A11)	Endothelial/PASMCs	Inhibits AMPK, activates mTOR promoting growth	Repurposed inhibitors (e.g., Sulfasalazine)
Immunoregulatory Macrophages	Immune Cells	Skew lung microenvironment from pro-inflammatory to regulatory	Cell therapy or agents to induce M2reg phenotype
TRP Channels	PASMCs	Mediate calcium influx in response to hypoxia/stretch	Specific TRP channel antagonists
Lung–Gut Microbiota	Systemic	Dysbiosis promotes systemic inflammation	Probiotics, targeted antibiotics, fecal transplant

**Table 3:** Selected Pharmacological Agents in Clinical Trials or with High Preclinical Potential for Hypoxic Pulmonary Hypertension.

Drug/Compound Class	Example Agent(s)	Mechanism of Action	Stage of Development	Key Findings/Potential
Prostacyclin Analogs/IP Receptor Agonists	Treprostinil, Iloprost, Selexipag	Vasodilation, anti-proliferative, anti-platelet	Clinical Use/Phase III	Approved for PAH; studied in PH associated with hypoxic lung diseases (e.g., ILD, COPD) for symptomatic relief and potentially improved exercise capacity.
Endothelin Receptor Antagonists (ERAs)	Bosentan, Ambrisentan, Macitentan	Blockade of ET-1 mediated vasoconstriction and proliferation	Clinical Use/Phase III	Approved for PAH; clinical trials in PH due to chronic lung disease have shown mixed results, with concerns about gas exchange worsening, but may benefit a subset of patients.
Phosphodiesterase-5 (PDE5) Inhibitors	Sildenafil, Tadalafil	Increase cGMP, enhancing NO-mediated vasodilation	Clinical Use/Phase IV	Approved for PAH; extensively studied in hypoxic PH (e.g., high-altitude, COPD). Shown to reduce PVR and improve exercise capacity, though long-term benefits on mortality are less clear in some patient groups.
Soluble Guanylate Cyclase (sGC) Stimulators	Riociguat	Sensitizes sGC to NO and directly stimulates it, increasing cGMP production	Phase II/III	Approved for PAH and CTEPH. Clinical trials in PH-ILD (e.g., RISE-IIP) were negative, highlighting the complexity of treating PH due to parenchymal lung disease. Further studies ongoing.
Rho-Kinase (ROCK) Inhibitors	Fasudil	Inhibition of ROCK, reducing vasoconstriction and vascular remodeling	Preclinical/Phase II	High preclinical potential. Shown to attenuate hypoxic PH in animal models by reducing vascular tone and remodeling. Early phase clinical trials in other forms of PH show promise.
Selective Serotonin Receptor Antagonists	Terguride	Blockade of 5-HT2A/2B receptors, reducing smooth muscle proliferation	Phase II	Demonstrated ability to reduce vascular remodeling and improve hemodynamics in experimental models of hypoxic PH. Clinical trials have been initiated but further development is needed.
Mitochondrial-Targeted Antioxidants	MitoQ, MitoTEMPO	Scavenge mtROS, improving mitochondrial function	Preclinical	High pharmacological potential. In animal models of hypoxic PH, these agents have been shown to reduce ROS, improve endothelial function, and attenuate pulmonary vascular remodeling.
Metabolic Modulators	Dichloroacetate (DCA)	Inhibits pyruvate dehydrogenase kinase (PDK), shifting metabolism from glycolysis to glucose oxidation	Preclinical/Phase I	Reverses the glycolytic switch (Warburg effect) in pulmonary vascular cells. Shown to reverse established PH in animal models by promoting apoptosis of proliferating cells.

## Data Availability

No new data were created or analyzed in this study. Data sharing is not applicable to this article.
